# Preclinical Evaluation of Novel SGLT2-Targeted Near-Infrared Optical Imaging Agent for Early-Stage Pulmonary Adenocarcinoma

**DOI:** 10.1007/s11307-025-02029-w

**Published:** 2025-07-14

**Authors:** Katherine Ortmeyer Welch, Kelly Anne McGovern, Lydia Chen, Ryan Krouse, Kevin Guo, Jeffrey Huang, Michael Brown, Jake Mlakar, Venu Bandi, David Holt, Paul Zhang, Sunil Singhal

**Affiliations:** 1 Division of Thoracic Surgery, Hospital of the University of Pennsylvania, Philadelphia, PA 19104 USA; 2 Department of Clinical Studies, University of Pennsylvania Veterinary Hospital, Philadelphia, PA 19104 USA; 3 Department of Pathology and Laboratory Medicine, Hospital of the University of Pennsylvania, Philadelphia, PA 19104 USA

**Keywords:** Non-small cell lung cancer, Molecular imaging, Optical imaging, Sodium-glucose cotransporter 2, Localized lung cancer

## Abstract

**Purpose:**

Lung cancer is increasingly diagnosed at early stages, but intraoperative localization of early lesions remains challenging. Intraoperative molecular imaging (IMI) aids in localization of tumors during surgery; however, no optical agents are targeted specifically for early-stage lesions. The sodium-glucose cotransporter 2 (SGLT2) has been implicated in early lung carcinogenesis. This study aimed to describe SGLT2 expression in early-stage lung adenocarcinoma (LUAD) and develop and validate a novel SGLT2-targeted near-infrared (NIR) contrast agent, GlucoGlo, for imaging LUAD.

**Procedures:**

SGLT2 expression was confirmed by immunohistochemistry (IHC) in human samples. GlucoGlo optical properties were characterized and compared to common NIR dyes. Sensitivity and specificity for SGLT2 were assessed using preclinical in vitro and in vivo mouse models.

**Results:**

On IHC, stage I LUAD displayed higher SGLT2 expression than stage II-III LUAD and normal lung tissue. GlucoGlo exhibited similar depth of penetration and resolution to FDA-approved contrast agents. SGLT2-expressing cell lines treated with GlucoGlo displayed higher fluorescence than the control cell line, confirming SGLT2-dependent uptake. Fluorescence increased with both incubation time and GlucoGlo concentration. Glucose and unconjugated GlucoGlo ligand competitively inhibited GlucoGlo in a dose-dependent manner, indicating high affinity and specificity. GlucoGlo selectively accumulated in SGLT2-expressing flank xenografts, with mean SBR of 2.23 and was inhibited by pretreatment with unconjugated GlucoGlo ligand.

**Conclusions:**

These findings support the potential of GlucoGlo as a targeted IMI contrast agent for early-stage LUAD, and they provide a foundation for future in vivo studies and translational development.

## Introduction

Lung cancer is the leading cause of cancer death worldwide, accounting for over 20% of cancer deaths [1]. Patients treated at early stages have the highest likelihood of cure, and fortunately, the increasing adoption of lung cancer screening and diagnostic computed tomography (CT) has led to more cancers being discovered at early stages [2, 3]. However, early-stage tumors are often challenging to resect as they lack significant parenchymal distortion or a solid component for visual or tactile identification. Consequently, resections are frequently delayed until nodules increase in size or density, and 10% of patients require intraoperative conversion to an open procedure for nodule localization [4–6]. Despite early treatment, up to 15% of patients with clinical stage IA lung cancer experience locoregional recurrence, underscoring the need for improved techniques in tumor localization and margin assessment [7].

Intraoperative molecular imaging (IMI) is recognized as a successful clinical adjunct for enhancing cancer visualization during surgery by utilizing targeted fluorescent contrast agents that selectively accumulate in tumors. Once the fluorescent contrast agent has collected in the tumor, a wavelength specific excitation laser and camera system are used to enhance visualization of the lesion [8]. It has been adopted for gliomas, ovarian cancer, breast cancer, and lung cancer for the purposes of improving tumor localization, assessing margins, and detecting synchronous disease [8–12]. Despite these advancements, no currently available contrast agent effectively targets precursor and very early-stage pulmonary adenocarcinomas, the most common subtype of lung cancer in the US. In 2024 alone, nearly 50,000 Americans were diagnosed with stage I non-small cell lung cancer (NSCLC) [13]. Our study sought to address this gap by developing an optical contrast agent designed specifically for intraoperative localization of early-stage pulmonary adenocarcinoma because it is one of the most challenging operations for the thoracic oncologic surgeon.

The overexpression of glucose transporters to sustain enhanced aerobic glycolysis, known as the Warburg effect, is a defining feature of early-stage cancer cells and offers opportunities for targeting [14]. This phenomenon has been exploited for cancer detection in positron emission tomography (PET), which targets the glucose transporter protein (GLUT) family commonly overexpressed in cancer cells. However, GLUT transporters are more commonly expressed on advanced cancers, and early-stage lung adenocarcinomas are often missed with this imaging modality [15, 16]. In contrast, emerging research has highlighted the role of the sodium-glucose cotransporter (SGLT) family in premalignancy and early cancer metabolism [17]. SGLTs, a group of sodium-coupled glucose transporters, primarily function in glucose absorption in the intestines and kidneys. Among these, SGLT2 has been shown to be upregulated in various cancers, such as pancreatic, prostate, breast, and lung cancer, making it a promising target for imaging early-stage lung adenocarcinoma [18–20].

In this study, we present the preclinical evaluation of GlucoGlo, a novel SGLT2-targeted near-infrared (NIR) optical dye designed for intraoperative detection of early pulmonary adenocarcinoma. Leveraging SGLT2's unique expression in early-stage malignancy, we aim to address the limitations of current imaging modalities and provide a foundation for future strategies to improve tumor localization and margin assessment for early-stage lung cancers.

## Methods

### Tissue Samples and Immunohistochemistry

To ascertain the expression of sodium-glucose cotransporter 2 (SGLT2) in human lung cancers, we first compared SGLT2 staining across an array of normal lung tissue and pulmonary adenocarcinomas using immunohistochemistry (IHC). A tissue microarray with normal lung tissue was obtained from US Biomax (LC1201b, Rockville, MD, USA). Additionally, 44 tissue samples were obtained from patients at the University of Pennsylvania between 2023–2024 who underwent pulmonary wedge resection or lobectomy for lung cancer. The University of Pennsylvania Institutional Review Board approved this study, and all subjects provided written informed consent for use of their tissues.

IHC staining for SGLT2 was performed through a process of deparaffinization, rehydration, and washings in xylene, graded alcohols, and distilled water. The samples were then placed in 10 mM citrate buffer at pH 6 with subsequent microwave antigen retrieval procedure and then incubated with purified anti-SGLT2 monoclonal antibody (ab58298, Abcam, Cambridge, MA, USA) at 1:1000 dilution. The antigen–antibody reaction was visualized using the avidin–biotin-peroxidase complex and diaminobenzidine as the chromogen. The slides were counterstained with hematoxylin.

Technical adequacy of the staining was validated by normal kidney and lung parenchyma as known positive and negative controls, respectively. Scoring was performed by a board-certified pulmonary pathologist. Staining of the tumor cells was scored based on the intensity of the staining as negative (0), mild (1 +), moderate (2 +), and strong (3 +) in 4 discrete regions of interest. Samples were analyzed under 5 ×, 10 ×, and 20 × objectives. 3 + strong staining was readily visualized under the 5 × objective, 2 + moderate staining was visible at 10 ×, and 1 + weak staining required the 20 × objective to visualize staining. Stroma and other cellular components apart from tumor cells were not scored or analyzed. Scores in the 4 regions of interest were then averaged for a composite score.

### Study Drug

To develop an SGLT2-targeted imaging agent, we hypothesized that a member of the SGLT2-inhibitor class of medications could be used as an effective ligand. SGLT2-inhibitors are an FDA-approved class of medication for type II diabetes mellitus that function as competitive antagonists highly specific for SGLT2 over other SGLTs. Multiple small molecule SGLT2 inhibitors were assessed for suitability for molecular imaging. Among these, dapagliflozin was selected as the small-molecule ligand as it has high specificity for SGLT2 over other isoforms of SGLTs (IC50 = 1.2 nM for SGLT2; 1400 nM for SGLT1), and was most amenable to conjugation with a fluorophore [17]. Dapagliflozin can be administered intravenously or orally and has a wide therapeutic range up to 500 mg per dose with minimal side effects in both diabetic and non-diabetic patients, offering an ample dosing range for clinical translation [21]. Dapagliflozin was manufactured in compliance with Good Manufacturing Practices (Astra-Zeneca, Hyderabad, India). The dapagliflozin molecule was modified to facilitate conjugation and was covalently bound to a modified indocyanine green (ICG) molecule, as described in Lansdell et al. [22]. The GlucoGlo solution was stored at − 20 °C in dimethyl sulfoxide. Before utilization, the frozen vials were thawed and diluted with methanol, phosphate-buffered saline (PBS) or culture media for the appropriate application.

### Spectral Analysis

Absorption spectra of GlucoGlo were measured at 5 μM concentration in methanol with a near-infrared (NIR) JASCO V-700 spectrophotometer (JASCO, Easton, MD, USA) using a dye-free vehicle sample for correction. NIR emission spectra were recorded using the same solution with a QM8075-11-C fluorescence spectrophotometer (Horiba Scientific, Ontario, Canada). Depth of penetration was compared to other common NIR contrast agents including ICG and pafolacianine using a tissue phantom. The 1% Intralipid tissue phantom was prepared by diluting 20% Intralipid (Sigma-Aldrich, Burlington, MA, USA) with deionized water [23]. 10 μM aliquots of each dye were placed in a capillary tube at various depths of Intralipid solution and were imaged with 785 nm excitation and 820 nm emission wavelengths using the Pearl Imaging System (LI-COR Biosciences, Lincoln, NE, USA). Signal to background ratios (SBRs) and full width at half maximum (FWHM) measurements were performed using ImageJ (NIH; https://imagej.nih.gov/ij).

### In Vitro SGLT2 Expression and GlucoGlo Binding

Several human non-small cell lung cancer (NSCLC) cell lines were obtained from American Type Culture Collection (Manassas, VA, USA), including H1299 (RRID:CVCL_B7N8), H1666 (RRID:CVCL_1485), and A549 (RRID:CVCL_A549) to evaluate GlucoGlo fluorescence in relation to SGLT2 expression in human cell lines. Cell lines were maintained in vitro using standard growth media (RPMI 1640 media supplemented with 10% fetal bovine serum (FBS), 1% glutamine, and 1% penicillin/streptomycin). Cell lines were regularly tested and maintained negative for Mycoplasma spp. and were cultured at 37 °C in 5% CO_2_ in a humidified incubator.

As SGLT2 expression has not yet been well characterized in common NSCLC cell lines, SGLT2 expression in H1299, H1666, and A549 was first quantified using flow cytometry with SGLT2 antibody staining to establish SGLT2 expression in positive and negative cell lines. Cells were cultured in 6-well plates with standard growth media for 24 h. Cells were then stained with monoclonal anti-SGLT2 antibody (ab58298, Abcam, Cambridge, MA, USA) for 1 h at 4 °C and washed three times with PBS to eliminate unbound ligand. Cells were stained with an Alexa Fluor 488-conjugated secondary antibody (ab150113, Abcam, Cambridge, MA, USA) for 15 min at room temperature, protected from light. Antibody binding was assessed using an LSR Fortessa X-20 flow cytometer (BD Biosciences, San Diego, CA, USA). Samples were analyzed using FlowJo software (Ashland, OR, USA).

Once SGLT2 expression had been quantified in each cell line, we then evaluated GlucoGlo binding and immunofluorescence in the same cell lines. Cells were cultured in poly-L-lysine coated 8-chamber slides (Thermo Fisher Scientific, Somerset, NJ) with standard growth media for 24 h, then incubated with 10 μM GlucoGlo for 1 h at room temperature and washed three times with PBS. Slides were mounted with ProLong Gold Antifade Reagent containing DAPI (Fisher Scientific, Waltham, MA, USA) and covered with a glass coverslip. GlucoGlo fluorescence was imaged using a Leica DM6 B fluorescence microscope (Leica Microsystems, Wetzlar, Germany). Relative immunofluorescence of GlucoGlo in the various cell lines was then compared to the relative SGLT2 expression in the cell lines as measured by antibody staining.

### Dose Response Curves and Incubation Time

Dose–response studies were conducted with GlucoGlo concentrations ranging from 0.1 to 10 µM. Time-course studies were performed with incubation times ranging from 5 min to 2 h, maintaining the same staining protocol. To quantify the binding curve for GlucoGlo, H1299 cells were cultured for 24 h and then treated with varying concentrations of GlucoGlo ranging from 1 nM to 1 μM for 1 h at 37 °C and washed three times with PBS. Flow cytometric analysis was performed to quantify GlucoGlo fluorescence, using red laser excitation (640 nm) and fluorescence detection in the APC-Cy7 channel (720–840 nm). GlucoGlo binding was quantified by measuring the median fluorescence intensity of the treated cells. Analyses were completed in triplicate and analyzed using FlowJo software.

### Competitive Inhibition

Competitive inhibition assays were performed to evaluate the specificity of GlucoGlo for the SGLT2 transporter using both unconjugated dapagliflozin and unconjugated glucose, SGLT2’s physiologic substrate. H1299 cells were seeded in the fashion previously described and treated with 10 μM GlucoGlo in the presence of excess unconjugated dapagliflozin (up to 1 mM) or free glucose (up to 300 mM). The same staining protocol was maintained, and GlucoGlo fluorescence was imaged using a Leica DM6 B fluorescence microscope and analyzed using Image J.

### Small Animal Tumor Model and Imaging

Flank xenografts were established in female nude athymic mice (Taconic Biosciences, Germantown, NY, USA) by subcutaneously injecting 2 × 10^6^ H1299 cells in 50 μL PBS and 50 μL Matrigel (Corning Life Sciences, Corning, NY, USA). Once the flank xenografts were palpable, GlucoGlo was administered via tail vein injection (0.05 mg/kg; n = 5). As a negative control, mice (n = 5) were pretreated with 5 mg/kg unconjugated dapagliflozin for 2 days prior to administration of GlucoGlo at 0.05 mg/kg. 48 h after GlucoGlo administration, mice were imaged with the Pearl Imaging System. SBRs were calculated by comparing mean fluorescence in tumors areas to that of the same area on the contralateral flank. The Animal Care and Use Committee of the University of Pennsylvania approved all animal study protocols, and experiments were conducted in compliance with the Guide for the Care and Use of Laboratory Animals.

### Image Analysis and Statistics

Image analysis was conducted with ImageJ. After correcting for distant background fluorescence, ROI software was used to quantify GlucoGlo fluorescence and background fluorescence to calculate an SBR. Statistical analysis was performed using GraphPad Prism 8 (GraphPad Software, San Diego, CA, USA). Fisher’s exact tests and chi-square tests were used for categorical variables where applicable. Unpaired student t-tests and ANOVA were used to compare means, where applicable. P values < 0.05 were considered statistically significant.

## Results

### SGLT2 is Expressed in Stage I Lung Adenocarcinomas More Than Normal Lung Tissue and Stage Ii-Iii Lung Adenocarcinomas

**Fig. 1:**
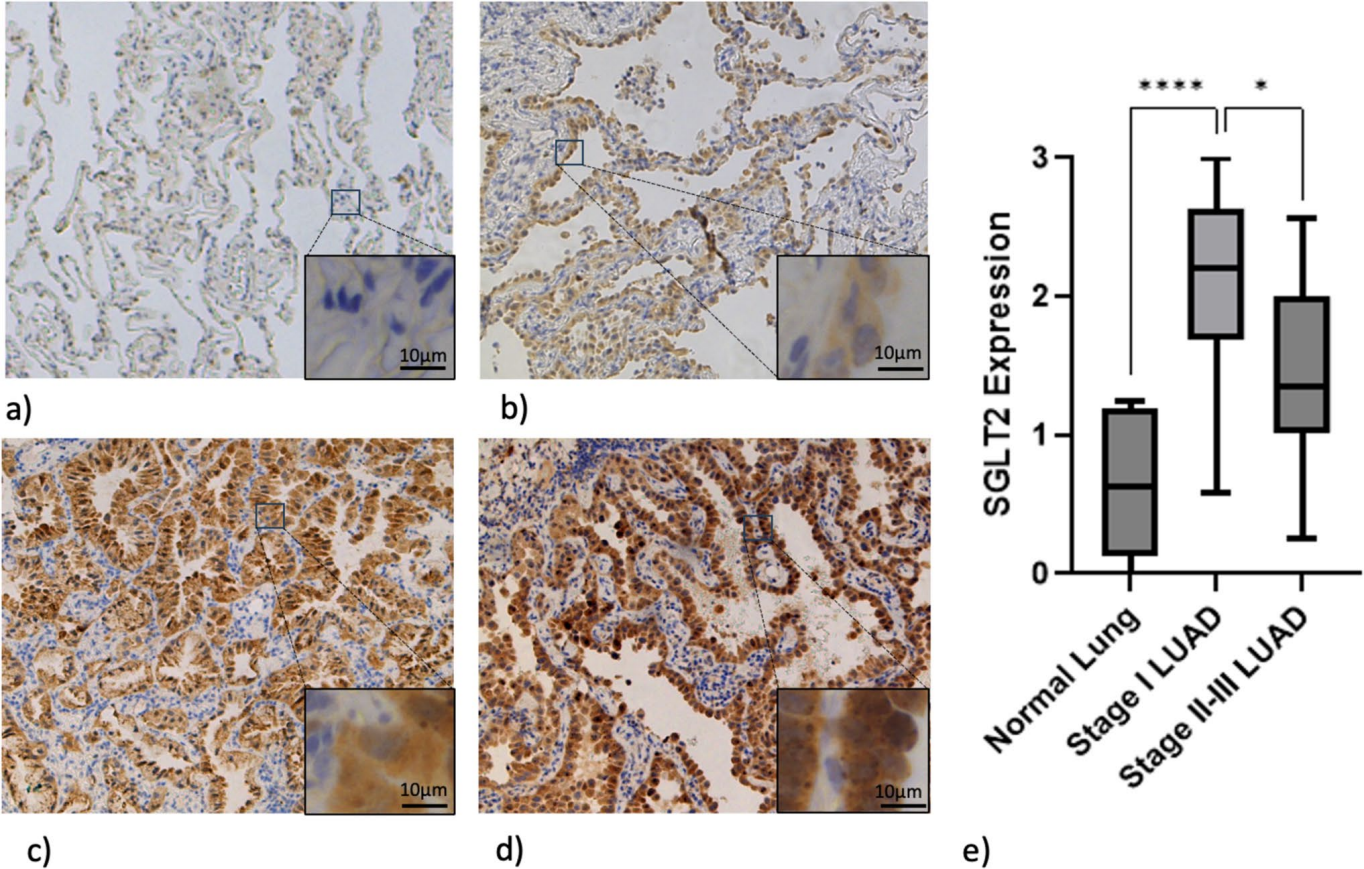
SGLT2 is expressed in stage I lung adenocarcinoma (LUAD) more than normal lung tissue and stage II-III LUAD. Representative staining of human lung specimens scored as 0, no staining (**a**); 1 +, weak staining (**b**); 2 +, moderate staining (**c**); and 3 +, strong staining (**d**), shown at 10 × magnification with 63 × inset. (**e**) Box and whisker plot demonstrates higher expression of SGLT2 in stage I LUAD compared to normal lung (p < 0.0001) and stage II-III cancers (p < 0.01).

We hypothesized that sodium-glucose cotransporter 2 (SGLT2) was a specific target for human lung adenocarcinomas (LUAD). To test this, we evaluated SGLT2 expression by immunohistochemistry (IHC) in LUAD across stages I-III and normal lung tissue (Fig. 1). Mean SGLT2 expression was significantly higher in stage I LUAD samples compared to normal lung tissue (2.08 vs 0.66, p < 0.001) and stage II-III cancers (2.08 vs 1.48, p < 0.01). We found that 90% (27/30) of stage I LUAD expressed SGLT2, with 23% (7/30) of the specimens showing a 1–2 + staining pattern and 66% (20/30) with 2–3 + staining. In contrast, normal lung tissue exhibited minimal SGLT2 expression, with 58% (7/12) of specimens showing no SGLT2 expression and 42% (5/12) of specimens having a 1–2 + staining pattern. Stage II-III LUAD also demonstrated reduced SGLT2 expression compared to stage I LUAD, with 25% (3/12) showing no SGLT2 expression, 42% (5/12) with 1–2 + staining, and 33% (4/12) with 2–3 + staining. These data confirm a high level of SGLT2 expression specifically in early-stage LUAD.

### GlucoGlo has Similar Optical Properties to Indocyanine Green

**Fig. 2:**
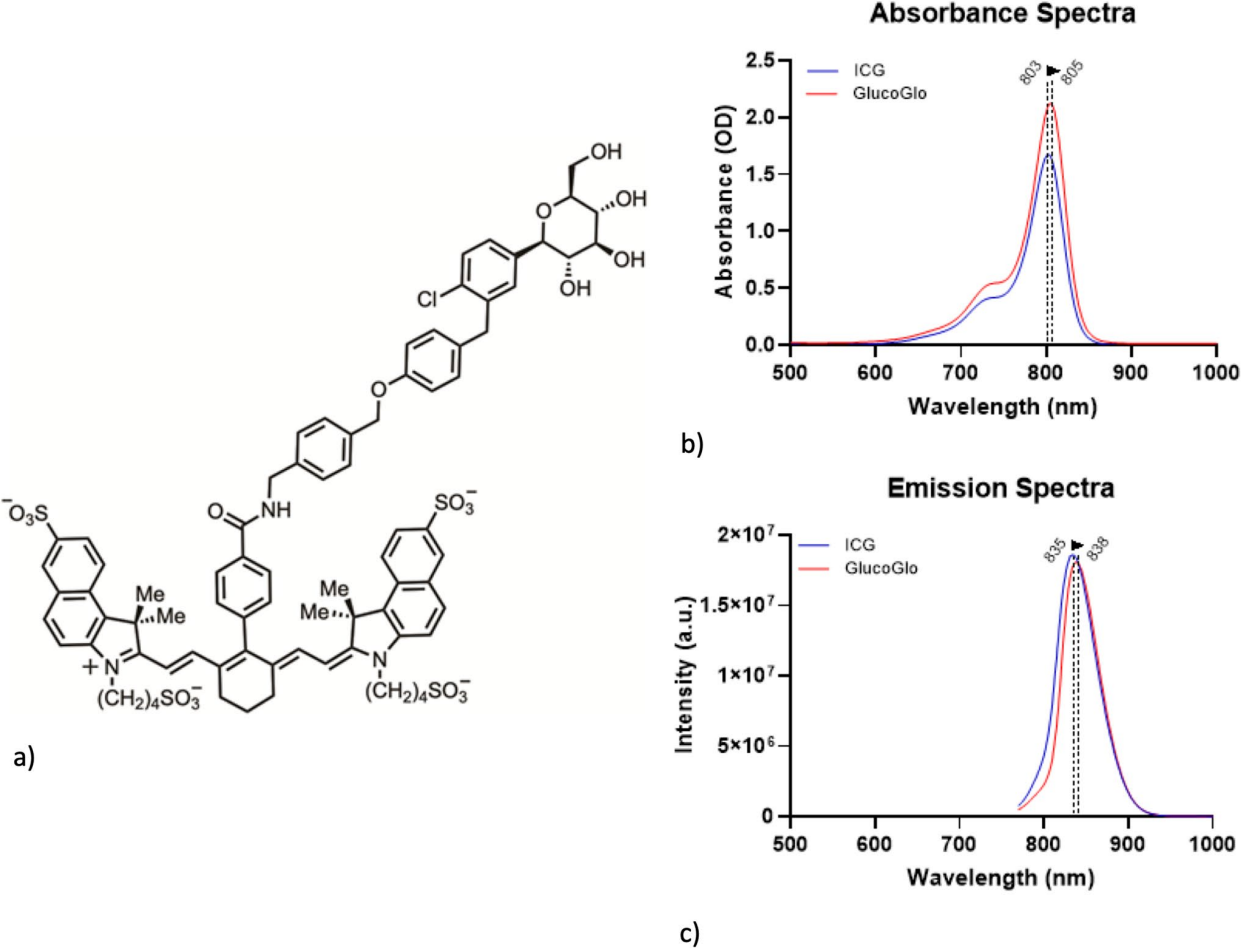
B**.** (**a**) Chemical structure of GlucoGlo. A modified SGLT2-inhibitor was covalently bound to a modified indocyanine green molecule to create GlucoGlo. The absorption peak and profile (**b**) and normalized emission intensity (**c**) for GlucoGlo were in the near-infrared range and were similar to the gold standard of NIR imaging, indocyanine green.

An SGLT2-targeted small molecule, dapagliflozin, was conjugated to a modified indocyanine green (ICG) molecule to create GlucoGlo (Fig. 2). To evaluate its compatibility with existing near-infrared (NIR) imaging systems widely used in US hospitals, we first characterized the optical properties of GlucoGlo to understand if they differed significantly from the base ICG. Spectroscopic analysis demonstrated that GlucoGlo underwent minimal shifts in absorption and emission compared to ICG. GlucoGlo had a peak excitation wavelength of 805 nm and a corresponding emission peak at 838 nm, closely resembling ICG’s excitation and emission peaks of 803 nm and 835 nm, respectively (Fig. 2). These findings confirm that conjugation of the ICG molecule with dapagliflozin did not significantly alter its fluorescent properties, making GlucoGlo well suited for NIR imaging applications.

### GlucoGlo Depth of Penetration and Resolution Matches Standard Clinically Available NIR Fluorophores

**Fig. 3:**
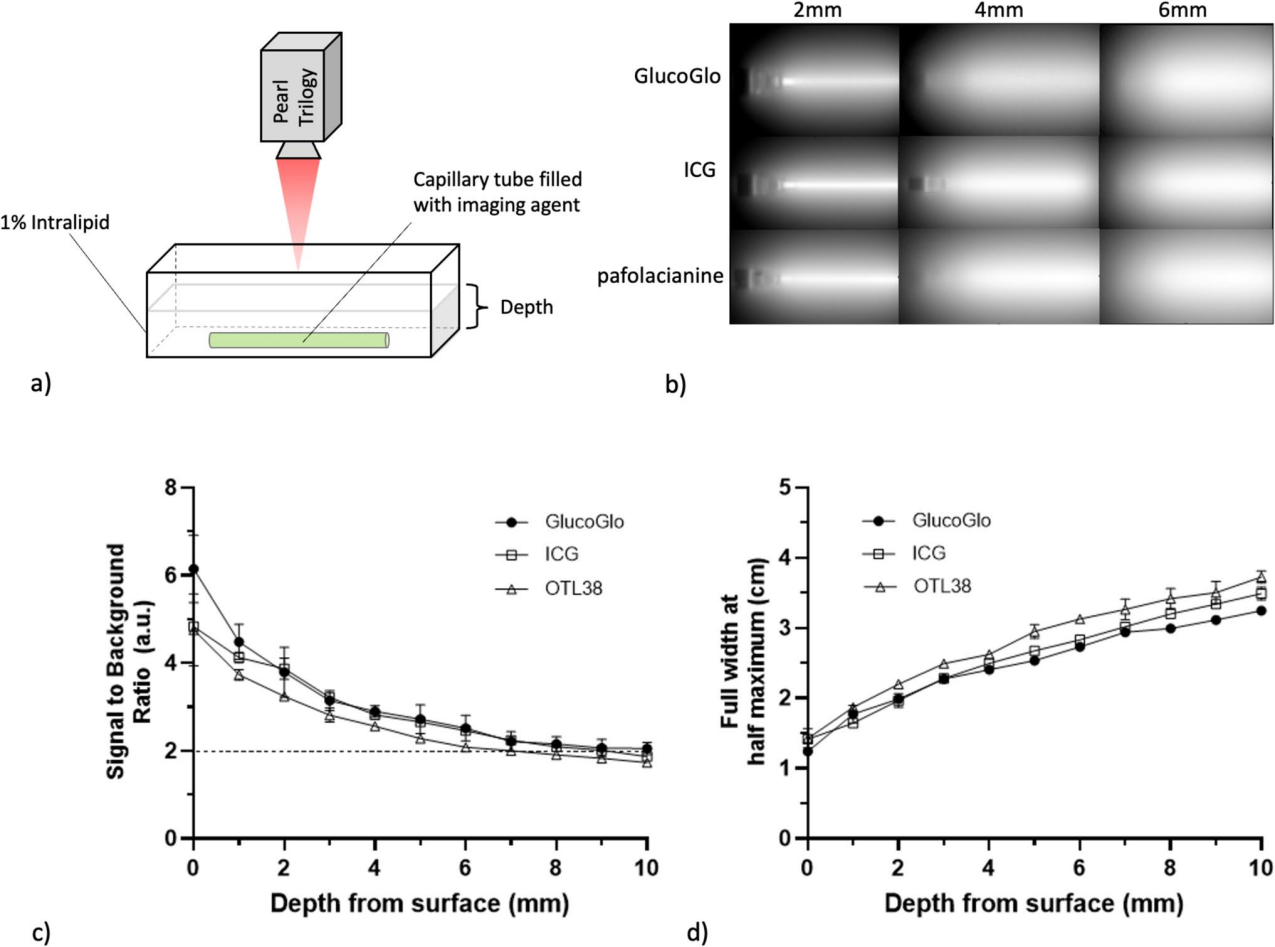
GlucoGlo depth of penetration and resolution matched standard clinically available NIR fluorophores. (**a**) Schematic diagram of 1% Intralipid tissue phantom. (**b**) Representative NIR images of GlucoGlo, indocyanine green (ICG), and pafolacianine at various depths of tissue phantom. (**c**) Signal to background ratios (SBR) of GlucoGlo at each measured depth of tissue phantom was comparable to ICG and pafolacianine. Dotted line represents the SBR threshold of 2, below which the signal cannot be reliably discerned from background fluorescence. (**d**) Full width at half maximum analysis of GlucoGlo, ICG, and pafolacianine at various depths of tissue phantom showed that GlucoGlo had a similar resolution to ICG (p = 0.15) and improved resolution compared to pafolacianine (p = 0.002).

We next compared the depth of penetration and resolution of GlucoGlo relative to currently available FDA-approved NIR optical agents, ICG and pafolacianine (a folate-targeted NIR tracer) using an intralipid tissue phantom (Fig. 3). SBRs were not significantly different between GlucoGlo, ICG, and pafolacianine at all measured depths. At a depth of 5 mm from the Intralipid surface, the SBRs for GlucoGlo, ICG, and pafolacianine were 2.72 ± 0.37, 2.65 ± 0.11, and 2.27 ± 0.07, respectively (p = 0.07). GlucoGlo SBRs decreased below the threshold of 2 at 8.27 ± 0.86 mm below the Intralipid surface, similar to ICG (8.30 ± 0.51 mm; p = 0.16) and at a greater depth than pafolacianine (7.43 ± 0.66 mm; p = 0.002).

To evaluate the resolution of the dyes, a FWHM analysis was performed at various depths for each dye. At 5 mm depth, GlucoGlo demonstrated similar resolution to ICG (2.53 vs 2.67 cm; p = 0.15) and had higher resolution than pafolacianine (2.53 vs 2.95 cm; p = 0.002). FWHM measurements increased with depth for all dyes and GlucoGlo displayed a slightly lower rate of increase compared to ICG and pafolacianine (p = 0.012).

### GlucoGlo Binds NSCLC Cell Lines in an SGLT2-Dependent Manner

**Fig. 4:**
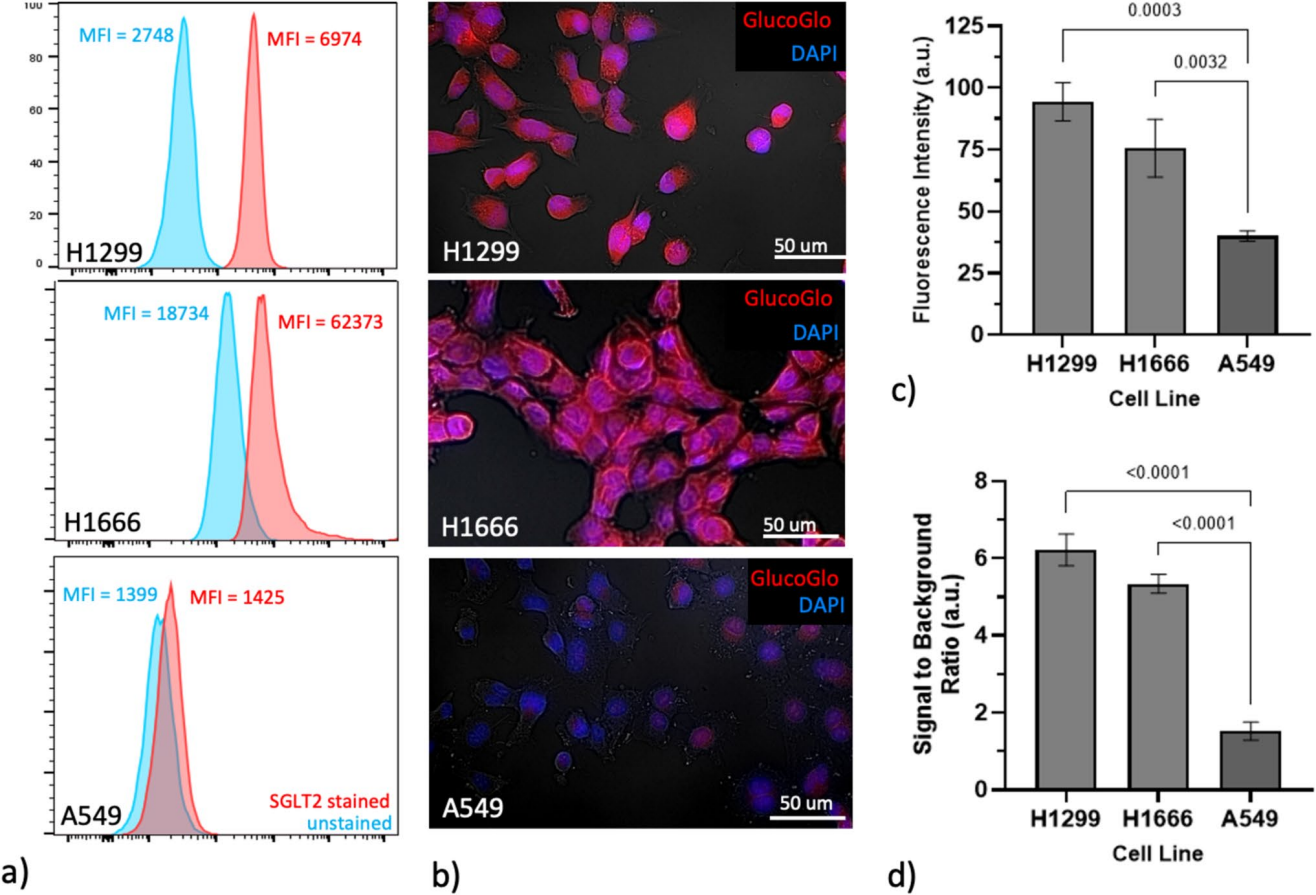
GlucoGlo binds models of human NSCLC in an SGLT2-dependent manner**.** (**a**) Representative flow cytometry tracings of cell lines after staining with SGLT2 antibody. Mean fluorescence intensity (MFI) of stained cells corresponds to the red histogram; unstained cells were used as a baseline (blue histogram). (**b**) Representative images of cell lines co-cultured with GlucoGlo (red pseudocoloration) and counterstained with DAPI (blue pseudocoloration) and examined by fluorescence microscopy, shown at 20 × magnification. (**c**) Fluorescence intensity (FI) and (**d**) signal to background ratios (SBR) of cells incubated with GlucoGlo were significantly higher in SGLT2-expressing cell lines compared to control lines measured by ANOVA (FI: p = 0.0065; SBR: p < 0.0001).

Next, we investigated whether GlucoGlo would bind SGLT2 on non-small cell lung cancer (NSCLC) models in vitro*.* Due to the limited existing data on SGLT2 expression in lung cancer cell lines, we first characterized the expression of SGLT2 on three representative NSCLC cell lines (H1299, H1666, A549). Immunostaining with an anti-SGLT2 monoclonal antibody revealed high SGLT2 expression in H1299 and H1666 cell lines and negligible expression in A549 cells, establishing A549 as a negative control cell line (Fig. 4).

Using SGLT2 expression as a baseline, we next analyzed GlucoGlo fluorescence in these cell lines using fluorescent microscopy. GlucoGlo fluorescence correlated with relative SGLT2 expression levels in each cell line. Strong fluorescent signal was present in H1299 and H1666 cells, while minimal fluorescence was observed in A549 cells (p = 0.0065). Similarly, SBRs of the SGLT2-expressing cell lines were significantly higher than non-SGLT2 expressing lines when incubated with GlucoGlo (p < 0.0001). Together, these results suggest that GlucoGlo binds human NSCLC models effectively and in an SGLT2-dependent manner.

### GlucoGlo Exhibits Dose-Dependent Binding to SGLT2 with Low Non-Specific Binding

**Fig. 5:**
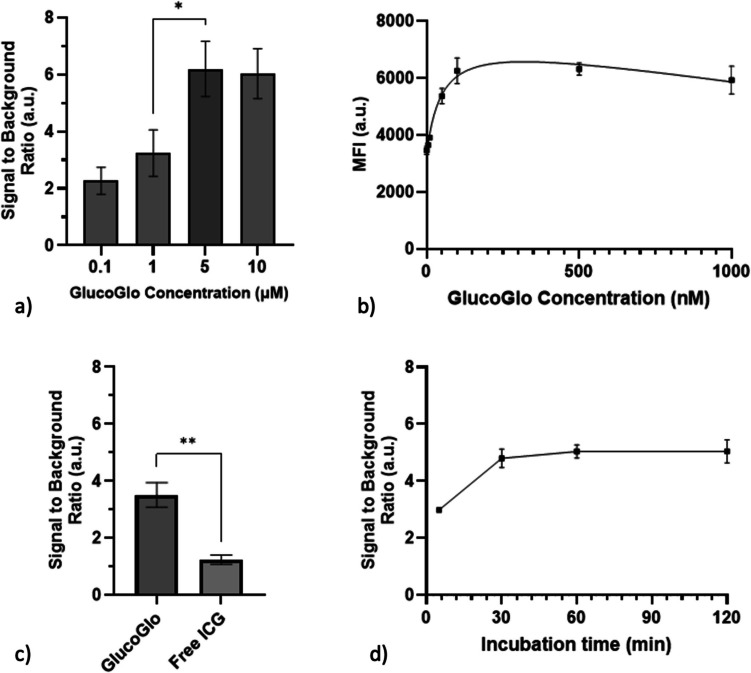
GlucoGlo binding increases with concentration and incubation time. (**a**) Signal to background ratios (SBR) of SGLT2-expressing cells incubated with GlucoGlo increased with dye concentration, plateauing at 5 μM with fluorescence microscopy (p < 0.05). (**b**) Flow cytometric measurement of GlucoGlo’s binding curve showed a strong binding affinity, with Kd = 50.25 nM. (**c**) Cells incubated with GlucoGlo had significantly higher SBRs compared to controls incubated with non-targeted ‘free’ ICG (p = 0.001). (**d**) SBRs increased with longer GlucoGlo incubation time (p < 0.0001), plateauing at 50 min.

In dose–response experiments, GlucoGlo fluorescence increased in a dose-dependent manner with increasing dye concentration in SGLT2-expressing cell lines (p < 0.05). SBRs plateaued at 5 µM, indicating an optimal concentration of 5 µM for in vitro imaging (Fig. 5). The negative control cell line, A549, maintained SBRs below the threshold of 2 at all tested concentrations, suggesting minimal off-target binding of the dye. Further supporting this specificity, H1299 cells incubated with GlucoGlo exhibited significantly higher SBRs compared to cells treated with the same concentration of free ICG, demonstrating that the SGLT2-inhibitor ligand, rather than the ICG moiety, is the primary binding component (p = 0.001). Binding curve measurements exhibited a dissociation constant (Kd) of 50.25 nM (95% CI: 27.89–87.79 nM).

In time-course studies, GlucoGlo fluorescence increased with longer incubation time in SGLT2-expressing cell lines. SBR values plateaued at 50 min, indicating that GlucoGlo binding reaches equilibrium with SGLT2 transporters by this timepoint. This suggests an optimal timeframe of 1 h incubation for in vitro imaging experiments to maximize signal intensity. Consistent with the dose response experiments, A549 cells did not exhibit substantial fluorescence at any tested incubation duration, further confirming the strong and specific binding of GlucoGlo to SGLT2.

### GlucoGlo Outcompetes Free Glucose for Binding to SGLT2

**Fig. 6:**
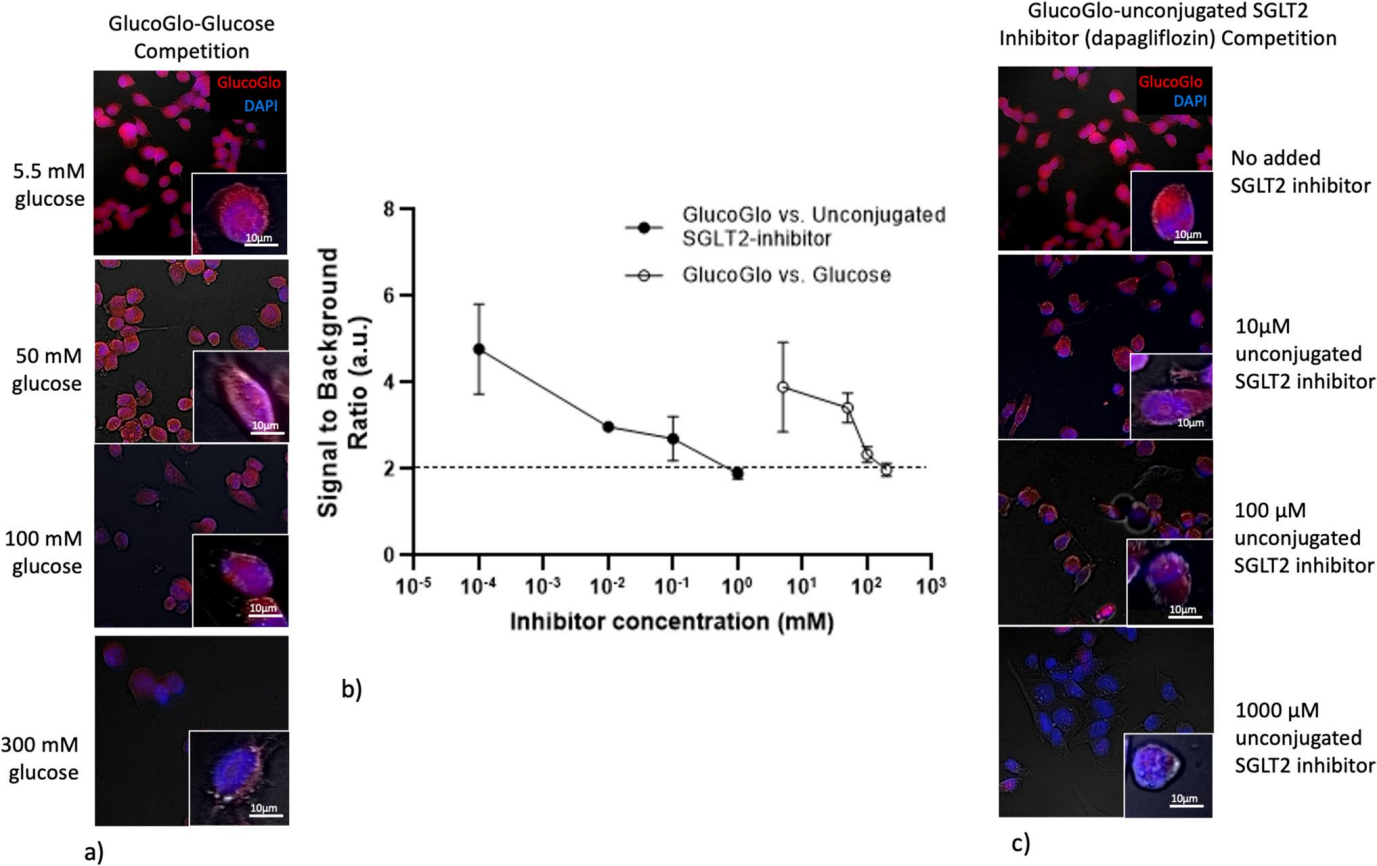
GlucoGlo is competitively inhibited by free glucose and unconjugated SGLT2-inhibitor. (**a**) Representative fluorescent microscopy images of H1299 cells showing progressively diminished GlucoGlo fluorescence with increasing concentration of glucose, shown at 10 × magnification with 63 × inset. (**b**) Signal to background ratios (SBR) of SGLT2-expressing cells decreased with increased administration of both competitive inhibitors, glucose and unconjugated dapagliflozin. Glucose required significantly higher concentrations than unconjugated dapagliflozin to decrease GlucoGlo fluorescence, demonstrating higher binding affinity of GlucoGlo for SGLT2 than glucose. (**c**) Representative fluorescent microscopy images of H1299 cells showing progressively diminished GlucoGlo fluorescence with increasing concentration of unconjugated dapagliflozin, shown at 10 × magnification with 63 × inset.

SGLT2 is a low-affinity transporter for sodium and glucose in humans. To investigate whether glucose could outcompete GlucoGlo for the SGLT2 transporter on cancer cells, we performed a competition assay with GlucoGlo and excess glucose. The starting glucose concentration was 5.5 mM, using low glucose cell culture media to approximate normal blood glucose levels in the human body [24]. At physiologic glucose levels, GlucoGlo binding to cells was readily visible by fluorescence microscopy (Fig. 6). GlucoGlo fluorescence diminished as free glucose concentration increased (p = 0.0013), confirming that GlucoGlo occupies the active site of SGLT2 and competes with glucose. Notably, high concentrations of glucose—approximately 30,000-fold excess relative to GlucoGlo—were required to reduce SBRs below 2, highlighting GlucoGlo’s strong affinity for SGLT2 over the transporter’s natural substrate.

We next performed a competition assay using excess unconjugated SGLT2 inhibitor, dapagliflozin. With increasing concentration of unconjugated dapagliflozin, GlucoGlo fluorescence diminished, confirming GlucoGlo’s specificity for SGLT2 (p = 0.027). Unlike with glucose, however, a 100-fold excess of dapagliflozin was sufficient to decrease SBRs below the threshold of 2, demonstrating an exponentially higher affinity of GlucoGlo and dapagliflozin for the glucose transporter. Together, these findings underscore GlucoGlo’s strong affinity and specificity for the SGLT2 transporter, validating its utility as a targeted imaging agent for SGLT2-expressing lung cancer cells.

### GlucoGlo Labels SGLT2-Expressing NSCLC Mouse Xenografts

**Fig. 7:**
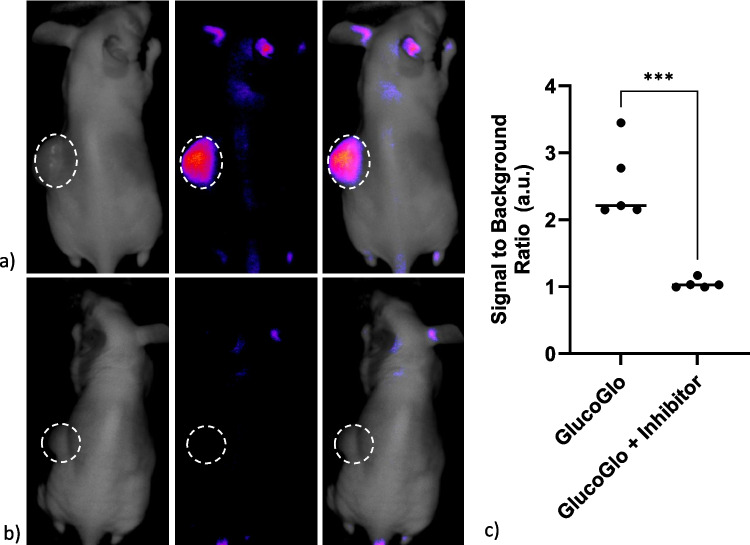
GlucoGlo selectively binds NSCLC flank xenografts in murine models. Representative brightfield, NIR, and overlay fluorescent images of NSCLC flank xenografts 48 h following administration of (**a**) 0.05 mg/kg GlucoGlo alone and (**b**) with the addition of 100-times the concentration of unconjugated dapagliflozin. White dashed circle denotes the tumor area. (**c**) Signal to background ratios (SBR) were significantly higher in tumor-bearing mice treated with GlucoGlo compared to those pretreated with 100-fold excess of dapagliflozin to GlucoGlo (5: 0.05 mg/kg; p = 0.0002).

To determine if GlucoGlo could label NSCLC in in vivo mouse models, female nude athymic mice bearing SGLT2-expressing H1299 flank xenografts were administered GlucoGlo at 0.05 mg/kg via tail vein injection. Mice pretreated with 5 mg/kg unconjugated dapagliflozin via tail vein injection for two days prior to GlucoGlo administration were used as a negative control. 48 h after GlucoGlo administration, tumors were found to have strong fluorescent signal compared to background fluorescence in the uninhibited group (Fig. 7). Pretreatment of the mice with unconjugated dapagliflozin eliminated the fluorescent signal from flank tumors, indicating SGLT2-specific binding of GlucoGlo. Mean SBR for tumors in mice administered GlucoGlo alone was 2.23 a.u., significantly higher than in mice pretreated with SGLT2 inhibitor (1.01; p = 0.0002), indicating excellent tumor-specific labeling.

## Discussion

In this study, we performed a preclinical evaluation of GlucoGlo, a novel sodium-glucose cotransporter-2 (SGLT2)-targeted near-infrared (NIR) probe designed for imaging of early-stage lung adenocarcinoma (LUAD). Our findings demonstrate that SGLT2 is significantly overexpressed in stage I LUAD compared to normal lung and later stage cancers. GlucoGlo has comparable optical properties to the gold standard for NIR fluorophores and has a similar depth of penetration and resolution in tissue models. GlucoGlo accurately and selectively labels SGLT2-expressing cells in both in vitro and in vivo preclinical models with minimal off-target binding and it outcompetes glucose, the physiologic substrate of SGLT2. Together, these findings establish GlucoGlo as a sensitive and specific probe for SGLT2, suggesting it has potential utility in intraoperative molecular imaging (IMI) for early-stage lung cancer.

The success of IMI relies on identifying a target with differential expression between malignant and benign tissue to optimize sensitivity and specificity of the probe. Our findings demonstrated significantly higher SGLT2 expression in stage I LUAD with decreased expression in later stage cancers and minimal expression in normal lung tissue. These results are consistent with other reported literature highlighting SGLT2 expression in lung cancers, as well as breast, pancreatic, and prostate cancer [18–20, 25]. Furthermore, other studies have suggested that SGLT2 appears to be uniquely expressed in well-differentiated adenocarcinoma exhibiting lepidic or papillary growth patterns, reinforcing its utility as a target for early lung malignancy [26].

Physiologically, SGLT2 has highly restricted expression, largely limited to the proximal renal tubule where it is responsible for the resorption of 85% of urinary glucose. This limited expression has been exploited therapeutically with SGLT2 inhibitors for diabetes management. These drugs bind SGLT2 with affinities in the nanomolar range and decrease urinary uptake of glucose without impacting other glucose transporters essential for normal cellular function [27]. GlucoGlo leverages this specificity for SGLT2 in preclinical NSCLC models, selectively labeling tumor in an SGLT2-dependent manner with minimal background fluorescence. In addition, its fluorescence intensity was reduced by competitive inhibition by both unconjugated dapagliflozin and the physiologic substrate of SGLT2, glucose, indicating its specificity for SGLT2. Importantly, competitive inhibition of GlucoGlo by glucose occurred at substantially higher concentrations than free dapagliflozin, demonstrating that GlucoGlo has a higher affinity for SGLT2 than does glucose. Significant decreases in fluorescence resulting from glucose inhibition occurred only at supraphysiologic concentrations, suggesting that GlucoGlo is compatible with both diabetic and non-diabetic patients, a key consideration for clinical translation.

Early lung cancer lesions, particularly ground-glass opacities, pose unique challenges during surgical resection. These lesions often lack visible changes or a solid component for tactile identification and can appear indistinguishable from normal lung tissue during resection, leading to difficulties in localization and margin assessment. While patients certainly benefit prognostically from timely resection of early lung cancers, 30–55% of patients experience recurrence from their early cancers, suggesting that there is significant room for improvement in resection [5]. IMI has emerged as a helpful technology that aids in localizing occult primary lesions, detecting synchronous disease and assessing margin positivity [8, 12]. In particular, the use of fluorophores in the NIR spectrum increases the depth of penetration of probes and decreases autofluorescence of benign tissue [28]. Our results demonstrate that GlucoGlo’s optical properties are comparable to ICG and improved from pafolacianine, the current gold standards in NIR fluorophores. GlucoGlo’s similarity to ICG also enables it to be detected by several FDA-approved imaging devices widely used in the US, making GlucoGlo easily implemented clinically. Human studies will be important to optimize the timing of GlucoGlo administration in a clinical setting, whether in the immediate preoperative setting or during clinic visits in the days leading up to surgery, as is standard with other approved imaging agents.

Given the high expression of SGLT2 in stage I LUAD, patients with ground glass pulmonary nodules and well differentiated lepidic histology are likely to benefit most from GlucoGlo imaging [29]. While CT can identify these lesions preoperatively, complementary use of methyl-4-deoxy-4-FDG (Me-4-FDG), a PET tracer taken up by SGLTs, may help to stratify patients whose tumors are SGLT2-avid and therefore suitable for GlucoGlo-guided surgery [30, 31]. These modalities could function synergistically, with Me-4-FDG serving as a noninvasive predictor of GlucoGlo efficacy. To address the reduced SGLT2 expression seen in more advanced LUAD, GlucoGlo could be combined with additional dyes targeting markers of later-stage cancer, such as folate receptor, annexin, or specific pH environments [32–34]. This multiplexed “cocktail” approach may provide a composite fluorescent signature to better characterize pulmonary nodules, which may be especially useful in patients with multifocal disease or limited pulmonary reserve to prioritize the resection of higher-risk nodules.

This study is limited to preclinical models. Ongoing studies are needed to further clarify the efficacy of the dye using in vivo models. Ultimately, clinical trials will be essential to assess the usefulness of the dye for human tumors and identify which subgroups may derive the most benefit from use of the dye.

## Conclusions

This study introduces an SGLT2-targeted NIR probe, GlucoGlo, as a novel optical agent for IMI of early-stage LUAD. GlucoGlo’s specificity for SGLT2, minimal off-target binding, and NIR optical qualities highlight its potential for clinical translation.

## Data Availability

The authors confirm that the data supporting the findings of this study are available within the article. Additional raw data were generated at University of Pennsylvania. Data supporting the findings of this study are available from the corresponding author SS on request.
